# Smoking cessation in people with multiple sclerosis: qualitative study on the current practices and barriers for delivering assistance from the perspective of healthcare professionals in Germany

**DOI:** 10.1136/bmjopen-2024-091950

**Published:** 2025-07-07

**Authors:** Alex Maximilian Keller, Claudia H Marck, Daniel Kotz, Barbara von Glasenapp, Christoph Heesen, Karin Riemann-Lorenz

**Affiliations:** 1 Institute of Neuroimmunology and Multiple Sclerosis, University Medical Center Hamburg-Eppendorf, Hamburg, Germany; 2 The Melbourne School of Population and Global Health, The University of Melbourne, Melbourne, Victoria, Australia; 3 Institute of General Practice, Heinrich-Heine-Universitat Dusseldorf, Dusseldorf, Nordrhein-Westfalen, Germany

**Keywords:** Multiple sclerosis, Smoking Reduction, QUALITATIVE RESEARCH

## Abstract

**Abstract:**

**Objectives:**

Smoking is a well-established risk factor that exacerbates multiple sclerosis (MS) progression and increases disease activity. Smoking cessation promotion practices of MS clinicians are not meeting the needs of people with MS (pwMS). This study aimed to explore the current practices and barriers faced by MS clinicians in Germany.

**Design:**

A qualitative study design, using semi-structured interviews and thematic analysis.

**Setting:**

Interviews with participants were held online, via telephone or face-to-face at our institute in Hamburg, Germany.

**Participants:**

We recruited eight neurologists and four MS nurses from hospitals, neurology practices and rehabilitation facilities in Germany via purposive and snowball sampling.

**Results:**

We identified 27 codes across four themes: (1) knowledge: the 12 participants demonstrated a satisfactory general knowledge of the negative impacts of smoking on MS (2) current practice: significant variability was reported in the current practices, with some clinicians providing detailed advice while others merely assessing smoking status without further advice or assistance. (3) Barriers: key barriers identified included limited consultation time, perceived lack of patient motivation and insufficient availability of resources, like information material, for effective smoking cessation support. (4) Needs and wishes: participants wished for specific smoking cessation courses to which they could refer patients, as well as information material to use during patient counselling.

**Conclusion:**

The study reveals considerable gaps in the consistency and comprehensiveness of smoking cessation support provided by MS clinicians in Germany. Addressing these gaps through targeted interventions, and improving the availability of information materials could enhance smoking cessation promotion for pwMS.

**STRENGTHS AND LIMITATIONS OF THIS STUDY**

- This study included multiple sclerosis (MS) clinicians with different professions (neurologists and nurses).
- Every important type of facility, where most people with MS are being treated, is evenly represented (hospital, rehabilitation facility, neurology practice).
- Chance for selection bias, as purposive sampling and snowballing were used in an existing network to recruit participants.
- Social desirability bias cannot be ruled out, as participants were asked about their personal current practice.

## Introduction

 Multiple sclerosis (MS) is a chronic autoimmune disease that affects the central nervous system. It can lead to a wide range of symptoms such as muscle weakness, coordination problems, fatigue or cognitive difficulties.^1^ The exact cause of MS is unknown, but it is likely that a combination of genetic and environmental factors can influence its onset.^2^ In 2023, there were 280 000 people with MS (pwMS) living in Germany.^3^

While there is no cure for MS as of today, medical and behavioural treatments are available to manage symptoms and slow disease progression.^4^,^6^ Smoking is a known and established risk factor that accelerates and increases disease progression and activity.^7 8^ This is also the case for passive tobacco smoking.^9^ PwMS who smoke also show a significantly higher burden of T2-lesions,^10^ an increased risk for depression and anxiety and a lower quality of life compared with people who do not smoke.^11 12^ Additionally, pwMS who smoke run a higher risk of developing antibodies that render MS medication less effective.^13 14^ Different studies have also hypothesised smoking as a relevant risk factor for MS onset.^8 15 16^ This is, however, not unchallenged, as a recent systematic review of Mendelian randomisation studies on MS could not identify evidence for the connection of smoking and MS onset.^17^

However, for people who already have MS, the relevant question is what they can do to improve their disease prognosis. It has been shown that important disease outcomes of pwMS who stopped smoking after diagnosis do not significantly differ from pwMS who never smoked^9^ and that smoking cessation effectively reduces the risk of reaching disability milestones early.^18^ Calls have been made to improve smoking cessation promotion for pwMS, including through better research on this topic.^19^ Yet, studies from German MS populations report prevalences of smoking between 19% and 24%,^10 20^ with the actual prevalence likely being even higher.

The German Society of Neurology explicitly advises clinicians to educate patients with MS about the negative effects smoking can have on the disease, as well as pointing them to possible treatments to successfully accomplish smoking cessation.^3^ Additionally, the Association of the Scientific Medical Societies in Germany^21^ recommends to all practitioners the use of brief intervention methods, like the 5 A’s model: Ask, Advice, Assess, Assist and Arrange; as well as referring them to further cessation support services.^22^ Yet, in primary care in Germany, only about 20% of people who smoke reported having received advice to quit from their practitioner and only 3.6% received an offer for therapy.^23^ Studies on people with MS also show that they rarely receive adequate support with their smoking cessation needs.^24 25^ This is unfortunate, as the delivery of behavioural interventions and the referral to further quitting services from clinicians has been shown to increase quit rates among people who smoke.^26^,^29^

It is unknown to what extent MS clinicians in Germany are aware of MS-specific consequences of smoking themselves and if they commonly assess smoking status, provide advice on the benefits of quitting and refer patients with MS who smoke to promote smoking cessation. We aimed to identify (1) whether MS clinicians in Germany were aware of the specific consequences of smoking on MS health outcomes and (2) current practices, barriers and facilitators among MS clinicians regarding the promotion of smoking cessation.

## Methods

The study’s report conforms to the Consolidated criteria for Reporting Qualitative studies.^30^

### Research team

Our research group comprised six individuals with expertise in neurology, health sciences, physiotherapy, psychology and public health. The interviewer (AMK) possessed prior experience in conducting and analysing interviews from unrelated projects in the field of health sciences. Prior to the study, no relationship was established between the participants and the interviewer.

### Participant selection

In order to recruit MS experts from different healthcare settings (hospitals, rehabilitation facilities and neurology practices) and occupations (neurologist, MS nurse), we purposefully chose and used connections from the broad German network of our working group, to get in first contact with some of our participants. In the period from April to July 2023, we initially approached 14 possible participants in total via email and recruited 11 of them. Two participants did not respond in the first place, and one further participant stopped responding to our emails before an interview could be conducted. In addition to our initial start, we also used a snowball system, by asking each participant for contact information for further possible participants. Through this, we managed to include one more participant. Hence, we recorded and analysed a total of 12 interviews.

### Setting

To account for an often-busy schedule of clinicians, we offered all our participants to conduct the interviews either via telephone, online or face-to-face. Finally, five interviews were held via telephone, and five via the data-protected online software *Webex* (V.43, Cisco Systems), while the interviewer (AMK) was in his office and the participants either at home or in their respective offices. Two interviews were held face-to-face in an office at our facilities at the University Medical Center Hamburg-Eppendorf. In all cases, the interviewer and the interviewee were the only ones present. The interviews were held in German and all quotes in this paper were translated into English in retrospect using the translation software DeepL. Additionally, all quotes were double-checked for fidelity by the main author and for comprehensiveness by all authors.

### Data collection

During April and May 2023, we developed a semi-structured interview guide based on a relevant previous study.^31^ The first draft contained six main questions with some additional optional questions. This draft guide was discussed internally within our working group, as well as with DK, CHM and two external experts with experience as practitioners. The gained feedback led to a revised version of the interview guide, now comprising seven main questions, some re-formulations and optional questions. The full interview guide can be found translated into English in the online supplemental materials.

Before the interviews, we asked all participants to fill out a short questionnaire, containing questions about demographics (age, sex, education, occupation), their professional background (years of experience in the field of MS, type of facility) and their self-perceived level of knowledge regarding MS and smoking. Additionally, everyone received detailed information about the design and the goals of the study and had to provide us with a written form of consent for their participation. We collected the questionnaire data and consent using the data-protected application of *LimeSurvey,* provided by the University of Hamburg. Participants were eligible to participate if they had a professional background as a neurologist or nurse with a special focus on MS. All interviews were conducted and audio-recorded in the time between May and July 2023. For transcription, we used the software *f4x* (by dr. dresing & pehl GmbH). The generated transcripts were manually revised and corrected by AMK. Concurrent to the conduction of the later interviews, analysis started for the first interviews. Data saturation was discussed between AMK and KR-L, and data collection stopped once it was perceived that no new themes arrived from the interviews.

### Theoretical framework

This research’s epistemology follows a realist approach. It assumes an unidirectional relationship between meaning, experience and language.^32^ Our research is mainly interested in the experience of MS clinicians and their way of practice in regard to smoking cessation support. Therefore, this approach allows us to focus on the individual experiences of experts and their expressions of such, without focusing too much on the assumption that meaning and experience are socially (re-)produced, as would be the case for a constructionist approach.^32^ Within this epistemological framework, we chose to carry out a theoretical and semantic thematic analysis. As the name suggests, this analysis looks for repeating patterns or themes within the data, which are commonly mentioned in one way or the other by different participants. The thematic analysis being theoretical means that we are focusing the analysis around the predetermined research questions. In our case, the focus lies on the current practice of MS clinicians and their awareness of the consequences of smoking on MS. Therefore, the analysis is driven by the interest of the researcher and allows for a more detailed description of the specific points of interest, rather than to try and describe all the data in overall less detail. This is further facilitated by staying on the semantic level for the analysis. The semantic approach does not try and look ‘for anything *beyond* what a participants has said’,^32^ but stays on the surface level, which allows us to describe the data of interest in detail, without opening the borders of interpretation too broadly.

### Data analysis

For coding and analysing our data, we used MAXQDA (V.2022). For the thematic analysis, we followed a six-step process, which was suggested by Braun and Clarke.^32^

In a first step, all the coders (AMK, KR-L and BvG) read all the transcripts several times to familiarise themselves with the data (step 1). For the second step, AMK analysed the first four interviews and generated a first set of 25 codes. KR-L then used the initial codes by going through the same four interviews, creating new codes or commenting on existing ones. Before moving on to the next interviews, AMK and KR-L discussed all codes and made comments together, condensing or expanding the set of codes if necessary. Finally, a set of 27 codes was defined (step 2). This set of codes was then used by all three coders to analyse all interviews. When all interviews were analysed, the coders organised the codes into thematically fitting categories. Like this, five categories were formed (step 3). With the codes and categories in mind, the coders checked all codes for coherence within the overall data structure. Discussion within the team was used to resolve any differences that came up during the coding process until all coders agreed that all codes were fitting and coherent (step 4). Based on the codes and categories, the coders then formulated four main themes, which encapsulated the main essence of the data and helped answer our research question (step 5). These themes and overall findings are reported in detail in the results section (step 6).

### Reporting of findings

Each quote presented in the findings includes the participant pseudonym (eg, CL001), the type of facility they are working in (H=hospital, R=rehabilitation, N=neurology practice) and the years of experience in the field of MS (eg, CL001, H, 23).

### Patient and public involvement

Patients or the public were not involved in the design, or conduct, or reporting, or dissemination plans of this research.

## Results

### Participants characteristics

All interviews lasted between 15 and 25 min, with a mean duration of 20 min. We interviewed 12 clinicians: 8 neurologists and 4 MS nurses. Three out of all 12 participants reported to be smokers themselves. All detailed participant characteristics can be found in table 1.

**Table 1:** Sample characteristics (N=12)

Variable	N (%) or median (range)
Sex	
Female	5 (42)
Male	7 (58)
Median age (range)	58 (50–65)
Profession	
Neurologist	8 (66)
Nurse	4 (33)
Type of facility	
Rehabilitation	4 (33)
Neurology practice	4 (33)
Hospital/clinic	4 (33)
Median working experience with MS in years (range)	25 (5–35)
Self-assessed knowledge about MS	
Low	0 (0)
Medium	1 (9)
High	11 (91)
Self-assessed knowledge about smoking	
Low	1 (9)
Medium	8 (66)
High	3 (25)

MS, multiple sclerosis.

### Themes

**Figure 1:**
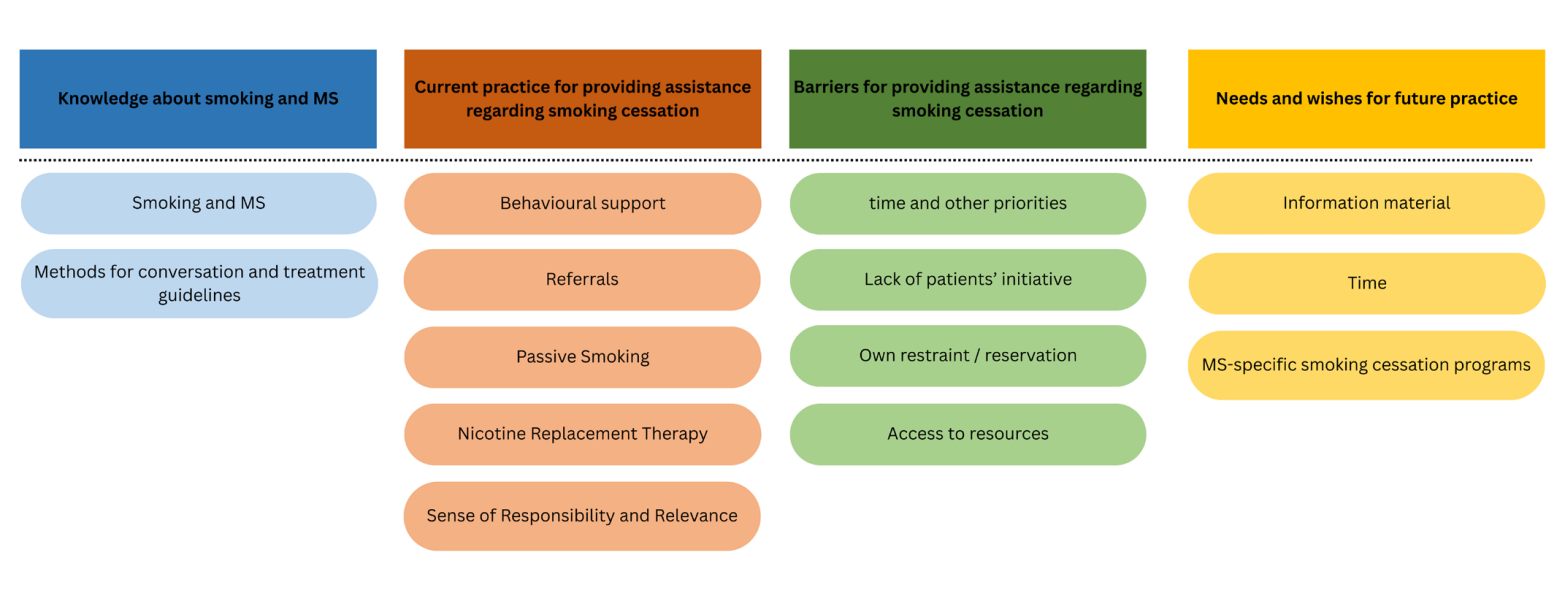
Overview of the four main themes with respective subthemes. MS, multiple sclerosis.

We identified four main themes, which encapsulated the main aspects discussed by our participants and which helped answer our research questions. Those four themes are (1) knowledge about smoking and MS, (2) current practice for providing assistance regarding smoking cessation, (3) barriers for providing assistance regarding smoking cessation and (4) needs and wishes for future practice. Following this, each theme and its subthemes will be explored in detail, and quotes from the interviews will be provided in separate tables. Figure 1 provides an overview of all four themes and their respective subthemes.

#### Knowledge about smoking and MS

We asked participants in our short questionnaire to self-assess their knowledge of MS and their knowledge on smoking. Further, we asked in the interviews what they know about the connection of MS and smoking and if they know any treatment guidelines or brief interventions for smoking cessation. Exemplary quotes for this theme and its subthemes can be found in table 2.

**Table 2:** Quotes for the theme ‘Knowledge about smoking and MS’

Quote	Subtheme
‘We know that smoking is harmful regarding the development of MS. So, it is a risk factor, both active and passive smoking, and that smoking is also harmful for the course of MS, both in terms of the occurrence of relapses and the occurrence of progression.’ (CL001, H, 23)	Smoking and MS
‘Well, I think there’s a relatively solid body of evidence that smoking negatively affects MS in many ways. The disease is more active, with the progression pace being faster depending on the stage of the disease.’ (CL003, N, 30)	Smoking and MS
‘I’m thinking…I’ve just been thinking whether smoking promotes relapse frequency or progression or is a risk factor for the onset of MS. But it should actually be a risk factor for relapses or for progression.’ (CL008, R, 25)	Smoking and MS
‘There are some doctors or maybe even psychologists with us who do that (use conversation guidelines). But I don’t know exactly whether it is done regularly. However, this is not a conversation guide that is implemented with us.’ (CL001, H, 23)	Methods for conversations and treatment guidelines
P1: ‘Do you personally know any current guideline recommendations for tobacco cessation?’ P2: ‘Not really. No. I admit.’ (CL006, R, 30)	Methods for conversations and treatment guidelines

MS, multiple sclerosis.

### Smoking and MS

The interviews mostly mirrored the self-assessment of the participants regarding their knowledge about MS and smoking. Despite eight people self-assessing their knowledge about smoking only as ‘medium’, most (n=10) participants very well knew about the connections of smoking and MS. They were able to describe, in varying levels of detail, the increased risk for MS onset and progression. Only a few (n=2) participants did not mention specific consequences of smoking on MS, but were still aware of the general negative contribution smoking can have towards the disease.

### Methods for conversations and treatment guidelines

We also asked our participants whether or not they know (or use) brief interventions (like the 5 A’s model: Ask, Advice, Assess, Assist and Arrange) or treatment guidelines specifically for smoking cessation. Here, no clinician knew or used specific methods for smoking cessation promotion. Only one participant explained that he would follow smoking cessation treatment guidelines by asking patients about their smoking behaviour.

#### Current practice for providing assistance regarding smoking cessation

We asked participants about their current practice and if and how they talk with their patients about the topic of smoking and MS. Exemplary quotes for this theme and its subthemes can be found in table 3.

**Table 3:** Quotes for the theme ‘Current practice for providing assistance regarding smoking cessation’

Quote	Subtheme
‘So, when I see the patient for the first time, for example, factors such as risk factors and risk behaviour are of course assessed, and smoking is also one of them. Depending on how it fits, this is then discussed, especially when it comes to the question of therapeutic influenceability.’ (CL004, R, 35)	Behavioural support
‘So, I basically always do that (raise the topic of smoking). That’s actually a standard sentence that I tell people that they shouldn’t smoke if possible.’ (CL007, H, 25)	Behavioural support
‘Yes, sure. So, as I said, we have a… If someone wants that, we have sufficient offers of help. We even have flyers in our waiting room about these offers of help.’ (CL007, H, 25)	Referrals
‘So, a strategy or leading the way… how to do it… that is not what I talk about with the patients.’ (CL003, N, 30)	Referrals
‘Yes, we do that occasionally (talk about passive smoking), although we don’t do it in a structured way for every patient.’ (CL001, H, 23)	Passive smoking
‘I’m not sure whether cessation therapies involving nicotine patches and similar approaches are truly effective.’ (CL003, N, 30)	Nicotine replacement therapy
‘Well, we basically have this responsibility (to talk about smoking) with all of our patients, not now with MS patients more than with all of our patients.’ (CL013, R, 31)	Sense of responsibility and relevance
‘And the data is relatively stable and has been confirmed several times by several research groups. So, it is highly relevant and probably also highly essential.’ (CL007, H, 25)	Sense of responsibility and relevance

MS, multiple sclerosis.

### Behavioural support

The quality and intensity of smoking cessation support as part of the current practice differed greatly among our participants. Some (n=6) participants explained that the topic of smoking is an integrated part of their routine, when talking with patients. In these cases, patients receive clear explanations about the consequences of smoking on their MS. One participant even reported to encourage people who do not smoke to not pick up smoking. Some (n=4) other participants mention the topic of smoking during their counselling, but without going into more detail with the patients about the MS-specific consequences smoking can have. Only two clinicians stated that they either do not talk about the topic with the patients at all or simply assess the smoking status without providing more information about the negative consequences of smoking on MS.

### Referrals

Some (n=5) of the participants refer the patients to further smoking cessation assistance services like courses or seminars, or provide flyers with details on where to seek further help. This was reported by all participants working in a rehabilitation facility, but only by one clinician working in a clinic. The remaining participants who work in either a clinic, hospital or neurology practice stated that they do not refer patients to cessation services or provide additional resources.

### Passive smoking

Only three participants reported discussing the topic of passive smoking and MS with their patients. However, they mentioned that this would not be part of a routine and therefore occurred only occasionally. All the other participants admitted to not speaking about passive smoking at all.

### Nicotine replacement therapies

Also, the possibility for patients to use nicotine replacement therapies (NRTs) when trying to stop smoking is discussed seldomly with patients by our participants. A few clinicians (n=3) reported discussing NRTs with patients who expressed interest. In contrast, clinicians who were uncertain about the effectiveness of NRTs (n=2) did not bring up this treatment option.

### Sense of responsibility and relevance

Many (n=8) participants acknowledged a high relevance of smoking in the field of MS and stated that the topic of smoking and MS should be actively addressed. They felt that it should be an integral part of conversations with pwMS about strategies to slow down progression and improve prognosis beside medical treatments. Only a few clinicians (n=3) considered smoking as less significant, compared with other topics, placing it in the ‘second row’ of priority. One participant doubted the scientific relevance of smoking and MS. In addition to the relevance of the topic, we also asked to what extent our participants feel responsible to talk about smoking and MS with their patients. Here, many participants (n=7) agreed that it is their clear responsibility as a practitioner, to not only mention smoking, but all other aspects that might be relevant for the patients’ health. Only one MS nurse said that she does not feel responsible to talk about smoking, as this would be the responsibility of the neurologists or physicians. A few (n=3) other clinicians additionally stated that, while they feel responsible to raise the topic of smoking and MS during consultations, they do not see themselves as smoking cessation agents. In their view, this responsibility is more appropriately placed with general practitioners.

#### Barriers for providing assistance regarding smoking cessation

We asked our participants what barriers exist that prevent them from providing (better) assistance regarding smoking cessation. Exemplary quotes for this theme and its subthemes can be found in table 4.

**Table 4:** Quotes for the theme ‘Barriers for providing assistance regarding smoking cessation’

Quote	Subtheme
‘It takes time to give good advice. And, of course, this is low in the medical field and not as high as one would like in other areas.’ (CL001, H, 23)	Time and other priorities
‘Time is just very short. And smoking is probably the smallest proportion.’ (CL007, H, 25)	Time and other priorities
‘And then the topic of nicotine is always something nice to have on top, but it usually falls behind, because everything else is higher in priority.’ (CL002, N, 25)	Time and other priorities
‘The second barrier is the patients’ comfort.’ (CL002, N, 25)	Lack of patients’ initiative
‘Some are very focused on doing something for themselves. Of course, it’s easy for them to follow the path. But there is also another group that says I don’t want to have anything to do with it for now, I just want to continue living normally.’ (CL004, R, 35)	Lack of patients’ initiative
‘And to be honest, I’m always a bit conflicted about it. On the one hand, for health reasons, you should of course stop and avoid it if possible. On the other hand, we often deal with people who are severely affected and who perhaps otherwise have no other joy in life and for whom that is the only thing they have.’ (CL006, R, 30)	Own restrain/reservation
‘So, the habit is certain, on the one hand… the biggest barrier is certainly me, that I don’t think about it and don’t find the time.’ (CL002, N, 25)	Own restraint/reservation
‘I would probably already have access if I searched online or whatever. But not like right away.’ (CL006, R, 30)	Access to resources
‘If someone wants that, we have sufficient offers of help. We even have flyers in our waiting room about these offers.’ (CL007, H, 25)	Access to resources

### Time and other priorities

The predominant barrier cited by participants when discussing the barriers in providing smoking cessation support to patients with MS was limited time. Only two participants reported having ample time availability, expressing no difficulty in addressing all important issues with patients. Conversely, many (n=9) others expressed frustration with their constrained schedules. Participants highlighted the wide range of topics they need to address during consultations, many of which compete for priority. Consequently, our participants often find themselves unable to talk more about smoking-related matters due to the pressing need to address medication and therapy concerns foremost. Moreover, the substantial time demands of administrative and organisational duties further limited the time allocated for counselling activities.

### Lack of patients’ initiative

We also got some (n=5) reports from our participants, who see the lack of patients’ initiative and motivation to change their health behaviour as an important barrier that prevents effective smoking cessation assistance. They explained that some patients have the expectation that it is the practitioner’s job to deal with the MS of the patients, while they can keep their health-related behaviours unchanged, including smoking. Other aspects mentioned in this matter were the perceived lack of patients’ acceptance and willingness to address the topic. Participants also said that patients often explain that smoking is closely linked to social settings, including daily routines or peer relationships, which makes quitting particularly difficult.

### Own restraint/reservation

We also had some (n=5) reports from our participants, who admitted that they restrain themselves from speaking more about smoking and MS. For example, clinicians expressed reluctance to raise the topic of smoking because they did not want to be perceived as moralising or acting as a scapegoat. A few (n=3) participants explained this in a bit more detail, saying that some pwMS already have to deal with a very difficult situation, for instance when they first receive their diagnosis. In these cases, our participants would refrain from advising them to stop smoking, to not overburden the patients with more challenges. Others would also not mention the topic of smoking if they perceived the patient to be generally unwilling to talk about it.

### Access to resources

We found that many (n=9) participants do not have any information material about smoking on MS or information for further smoking cessation services on hand. Four clinicians mentioned that they would probably find some information online if they were to look for it, but do not have it ready when talking with patients. Only a few (n=3) participants stated that they have materials like flyers or leaflets with information on the topic or with contact information of cessation services.

### Needs and wishes for future practice

Finally, we asked our participants what they would like to see change in the future regarding their practice on smoking cessation. Exemplary quotes for this theme and its subthemes can be found in table 5.

**Table 5:** Quotes for the theme ‘Needs and wishes for future practice’

Quote	Subtheme
‘So, I think such structured patient education programs, including digital health applications, apps,(…) and things like that, are extremely helpful.’ (CL003, N, 30)	Information material
‘Then I would wish that I actually had something on hand to give to the smokers when I came into contact with them.’ (CL011, H, 20)	Information material
‘I don’t know, maybe these numbers exist, but I’m not familiar with them at the moment. And that would actually be helpful in order to be able to estimate a little bit the importance of the effectiveness or the effect sizes of, um, of stopping, so to speak, or of quitting smoking. And I think if we had the numbers in black and white or knew how we could reduce the relapse rate by so many per cent, then we would probably pursue this more consistently. So, I find a comparison of the effect on the frequency of relapses extremely helpful.’ (CL008, R, 25)	Information material
‘And there is no separate program for… smoking in MS and I would very much welcome that if there was a smoking cessation program for patients and this also necessarily includes education, that is, a program for training regarding the consequences of smoking MS, both for doctors and patients.’ (CL001, H, 23)	MS-specific smoking cessation programmes
‘So structured program specifically for MS patients, that is, something that is tailored to MS patients. And this particularly includes the effects of smoking on the development and development of MS, because this does not occur in the normal smoking development program.(…)But I think that needs specific ones for MS. So, I would really like that.’ (CL001, H, 23)	MS-specific smoking cessation programmes
‘More time, staff, adequate compensation. Exactly.’ (CL006, R, 30)	Time
‘So, I would say that of course it plays a role how much time you have for people. And of course you could definitely wish for more time for lifestyle advice. That’s what people are demanding.’ (CL007, H, 25)	Time

MS, multiple sclerosis.

### Information material

Many (n=8) of our participants wished for more and better information material, which they can use in patient counselling. This includes both information about further referral services for smoking cessation, like hotlines, websites or courses; as well as summarised information about the latest studies on the topic of smoking and MS. Some (n=4) participants explained that this kind of information in the form of flyers or leaflets would help them significantly with the communication with patients. They perceived that using concrete figures or graphs for visualisation could facilitate their talking points during counselling.

### MS-specific smoking cessation programmes

A few (n=3) participants mentioned explicitly that they would welcome smoking cessation programmes that are tailored to the needs of pwMS. One clinician explained that the generic smoking cessation courses do not talk about how smoking can influence MS onset and progression, which is why an MS-specific smoking cessation programme could be useful. Here, participants mentioned various ways on how a programme like this could look like. In-person or online programmes in groups or fully digital platforms were mentioned.

### Time

Finally, despite time being one of the main barriers influencing the current practice of our participants, only two explicitly mentioned this aspect when asked what they would like to see to change in the future. Next to time, one participant also wished for more staff to tackle the various everyday tasks during clinical practice.

## Discussion

In Germany, smoking rates are high compared with other Western countries, including among pwMS.^10 20 33^ Smoking cessation is crucial to optimise health outcomes for pwMS,^18^ but research has shown that their needs for assistance with smoking cessation are currently not met.^24 25 34^ This study aimed to understand from the MS clinicians’ point of view, their practice, barriers and facilitators towards promoting smoking cessation. We found that the general knowledge of our participants about the effects of smoking on MS was satisfactory. Most participants mentioned the negative impact on disease progression and activity, as well as the higher risk for MS onset, while some others were a bit less specific. These findings are in line with an Australian study, also investigating (among other things) the level of knowledge among MS clinicians regarding smoking and MS.^31^ None of our participants, however, knew about or used conversation methods suitable for cessation support, such as the 5 A’s model (Ask, Advice, Assess, Assist and Arrange) or the ABC model (Ask, Brief advice, Cessation support). An increase in the usage of evidence-based brief interventions would be desirable, as a German study indicated that patients had increased odds for reporting a quit attempt, if they had received systematic advice from their general practitioner on smoking cessation, following either the 5 A’s or the ABC model.^28^ This increase is also proven to be feasible, since a 3.5 hours of training for clinicians for the usage of these models can increase the frequency of delivery of smoking cessation advice.^35^

The current practice of smoking cessation support was inconsistent among participants, in line with the aforementioned Australian study.^31^ While some explained that they inform their patients about the negative effects smoking can have on MS, others just mention it in less detail or simply assess the smoking status of their patients. So, in a lot of cases, the topic of smoking cessation is not discussed properly. These findings are in line with a German study which reported that nearly 81% of people who smoke from the general population who consulted either their general practitioner or a practitioner from a different medical field did not receive the advice to stop.^23^ Our own work, and others, have shown that people with MS report that their needs are not met when it comes to smoking cessation support.^25 34^ The lack of quitting advice could partly be explained by the barriers, which were mentioned by our participants. Here, a lack of time and perceived lack of motivation and initiative from the patients’ side were mentioned frequently. These two aspects were also identified to be most common by a systematic review, looking at barriers to the provision of smoking cessation interventions in an inpatient setting.^36^ However, according to German treatment guidelines, it should be the physician who actively mentions the topic of smoking during conversation with the patients as they should not rely on the patients initiative to make the first step.^22^

Within our sample, both passive smoking and the usage of NRTs were barely mentioned during patient counselling. Some of our participants explained this by simply not having thought about these topics regarding smoking cessation before. For passive smoking, most of them admitted that it should be probably discussed and talked about more frequently and systematically with pwMS. This would be desirable, as passive smoking causes similar negative effects on MS health outcomes as active smoking^9^ and should therefore be treated with similar seriousness and priority. Of note, a few participants reported that they would not recommend NRTs to patients as they hold the personal belief that they are not effective. However, a meta-analysis which included 70 randomised controlled trials examining NRTs versus control interventions showed the effectiveness of NRTs on successful smoking cessation.^37^ This gap in knowledge among clinicians needs to be addressed to improve the promotion of effective smoking cessation strategies.

While all our participants working in a rehabilitation facility offered referrals to smoking cessation services to the patients, it looked quite different for the participants working in a hospital setting or a neurology practice. This might be due to a different perception of responsibility, as rehabilitation facilities already focus on possible ways to help the patients overcome their direct and indirect disease burdens, while clinical settings might focus more on the diagnosis and medical treatment of the disease. But apart from that, we did not find any noteworthy differences based on profession or type of facility. Some participants also mentioned that they do not have referral or information material on hand to give to patients which further facilitates this problem. These findings are very similar to an Australian study investigating the current practice of MS clinicians.^31^ Our participants seemed to recognise this problem, as they commonly wished for better information material which they can use in the dialogue with patients, including information on referral services for smoking cessation. The facilitation of better referrals to smoking cessation services from clinicians could be an important step for pwMS. Proactive referral to smoking cessation programmes by healthcare staff can increase enrolment rates and therefore increase the chance of people quitting smoking.^38^

### Limitations

This study has some limitations that must be acknowledged. First, the use of purposive sampling and snowball techniques, while practical, may introduce selection bias, as participants were primarily recruited through existing networks. But we expect this effect to not be significant, as type of facility, geographical location and other participant characteristics are still well distributed. Still, future studies might want to consider including more sample characteristics (eg, ethnicity) to further ensure high heterogeneity among participants or to use a different sampling technique. Second, social desirability bias cannot be fully ruled out, as the interviews dealt with clinicians’ current practices, potentially leading participants to portray their practices in a more favourable light and not fully admitting to areas where their practice might be lacking. Therefore, our findings may be an underestimate of the current gaps in smoking cessation promotion among MS clinicians, further enhancing our conclusions. Finally, in some instances, a delay of 1 or 2 days occurred between the completion of the questionnaire and the interview. This could have introduced a potential bias, as we were unable to control whether participants informed themselves about the topic in the meantime.

### Conclusion

This qualitative study highlights the current practices and barriers faced by MS clinicians in Germany regarding smoking cessation promotion among their patients. The findings indicate that while most clinicians are aware of the negative impact of smoking on MS progression, there is a significant variation in how this knowledge is applied in clinical practice. Barriers such as limited time, lack of patient motivation and insufficient availability of resources for smoking cessation support were identified. The study underscores the need for targeted interventions, including the provision of comprehensive training for healthcare providers, as well as developing and providing them with information materials about the evidence base on smoking and MS and about referral pathways to smoking cessation services. By addressing these barriers, it should be possible to enhance the support provided to pwMS in their efforts to quit smoking, ultimately improving their health outcomes.

## Supplementary material

10.1136/bmjopen-2024-091950 online supplemental file 1

## Data Availability

Data are available upon reasonable request.
