# Supplementary material for: Smoking cessation in people with multiple sclerosis: qualitative study on the current practices and barriers for delivering assistance from the perspective of healthcare professionals in Germany
Source: BMJ Open. 2025 Jul 7;15(7):e091950. doi: 10.1136/bmjopen-2024-091950 (PMC12258274; doi:10.1136/bmjopen-2024-091950)
Supplement: online supplemental file 1 [file bmjopen-15-7-s001.docx]

| Supplementary Table 1: Interview Guide | | |
| --- | --- | --- |
| Leading question | Check (Was it mentioned? If not, ask the following questions) | Follow-Up Questions |
| Part 1: Literacy | | |
| Please tell me what you know about the connection between smoking and MS. | Do you feel well and adequately informed?  Are there MS-specific correlations, consequences, or disadvantages?  How relevant do you consider the topic of smoking in MS and educating patients about it?  Do you have any unmet needs for information on the topic of smoking and MS?  In your opinion, what is the most effective method to quit smoking?  Are you aware of current guideline recommendations for smoking cessation? (AWMF S3 Guideline on Smoking and Tobacco Dependence) | How do you inform yourself about the topic of smoking and MS? |
| What do you know about the consumption of cannabis among pwMS? |  |  |

| Part 2: Current Practice | | |
| --- | --- | --- |
| Tell me how and in what ways you counsel pwMS on the topic of smoking. | Are these practices theoretical, or are they actually applied and implemented?  If nothing is done: What are the reasons for this?  Do you make patients aware of specific support services such as smoking cessation programs?  Do you recommend nicotine replacement products?  When or how often is a patient's smoking status queried?  Do you also discuss the topic of passive smoking?  Do you use a specific conversation method for smoking cessation counseling? If so, which one? (e.g., 5 As or ABC).  How high is the demand for informational sessions? | Does the cannabis use of the patients also play a role in the counseling?  Do you also explicitly ask the patients about their cannabis use?  Do you also prescribe cannabis or cannabis products? |
| Part 3: Resources and Barriers for Referrrals | | |
| Which barriers exist that make patient counseling on smoking and MS more difficult for you?  In your opinion, are there aspects of patient counseling on smoking and MS that are already going well? | Do you have access to information or contact details for smoking cessation counseling, hotlines, contact points, or informational materials on the topic?  Is there time pressure from other important matters/issues when it comes to counseling on smoking?  Do the lack of financial incentives and compensation play a role?  Would you say you have a responsibility towards your patients to address the topic of smoking in MS? If not, in your opinion, who then has the responsibility? | What do you wish for in order to be able to offer good and effective smoking cessation counseling? |

| Part 4: Others | | |
| --- | --- | --- |
| Based on your experience, what characteristics do patients exhibit that contribute to successful smoking cessation?  Do you smoke yourself?  Are there any other aspects that haven't been mentioned yet that you would like to add? |  |  |
