# Supplementary material for: Smoking cessation in people with multiple sclerosis: qualitative study on the current practices and barriers for delivering assistance from the perspective of healthcare professionals in Germany
Source: BMJ Open. 2025 Jul 7;15(7):e091950. doi: 10.1136/bmjopen-2024-091950 (PMC12258274; doi:10.1136/bmjopen-2024-091950)
Supplement: Supplementary file 2 [file bmjopen-2024-091950.reviewer_comments.pdf]

PEER REVIEW HISTORY

BMJ Open publishes all reviews undertaken for accepted manuscripts. Reviewers are asked to complete a checklist review form and are provided with free text boxes to elaborate on their assessment. These free text comments are reproduced below.

ARTICLE DETAILS

Title (Provisional)

Smoking Cessation in People with Multiple Sclerosis: Qualitative Study on the current practices and barriers for delivering assistance from the perspective of healthcare professionals in Germany

Authors

Keller, Alex Maximilian; Marck, Claudia H; Kotz, Daniel; von Glasenapp, Barbara; Heesen, Christoph; Riemann-Lorenz, Karin

---

VERSION 1 - REVIEW

**Reviewer**	**1**
**Name**	**Buttolph, Lita**
**Affiliation**	**National University of Natural Medicine**
**Date**	**09-Sep-2024**
**COI**	**None**

Thank you for the opportunity to review this manuscript exploring the perceptions of MS clinicians on current practices and barriers to smoking cessation for patients with MS. The study highlights an important need for greater education and resources for MS clinicians regarding smoking education for patients in Germany (but likely relevant to other countries). I did not encounter any significant problems with this manuscript. I recommend publication of the manuscript with some minor editorial corrections listed below:

Line 25: Define “pwMS”

Line 163: Add “us” after “allow”

Lines 179, 253, 261, 265, 277, 278, 299: Remove the comma before the “that”

Lines 301-303: The sentence has some grammatical errors and needs more clarification regarding “social aspects.”

**Reviewer**	**2**
**Name**	**Polick, Carri S**

**Affiliation**	**Duke University**
**Date**	**26-Oct-2024**
**COI**	**No competing interests.**

Thank you for the opportunity to review this manuscript titled: Smoking Cessation in People with Multiple Sclerosis: Qualitative Study on the current practices and barriers for delivering assistance from the perspective of healthcare professionals.

This manuscript is timely and important. It will be a great addition to the growing body of research advocating for improved cessation services for people with MS. However, I recommend some edits before publication. The aim of this reviewer's comments is to give constructive feedback to help improve clarity, transparency, and the reader experience. Notably, it may first appear to be a lot of feedback, but I believe it is all very addressable.

Abstract

1) There is inconsistency while referencing interdisciplinary participants. The abstract clearly lists all participants as clinicians using parentheses, however, Table 1 then lists 8 clinicians vs 4 nurses. This introduces confusion in results when authors report things such as "only a few of the clinicians" because readers don't clearly know the denominator (e.g., 8 or 12?). I suggest being consistent throughout and when referencing only physicians, to use a more specific term like physician or neurologist to improve clarity.

2) Authors mention four themes and only report results on three. While the 4th can be somewhat inferred from the barriers, it would be more complete and balanced to clearly state the reported needs and wishes. Additionally, the last sentence of the results appears to report on theme 2 (current practices), but is listed under theme 3 (barriers). It would be clearer to list results together by theme, if this sentence is included after revisions, it should be moved up.

3) It appears that there is more than a slight risk of selection bias from using purposive/judgement sampling which relies on the judgement of researchers and is subject to additional (subconscious, conscious) bias. To improve transparency, this risk should just be openly acknowledged and "slight" should be removed. While this is also listed in the limitations, there is no mention of additional diversity of participants and particularly race or ethnicity. If future work could improve upon this work by broadening to a more diverse sample, it should be noted in the discussion or limitations.

Introduction

4) Line 82 – This may have a citation that was not included in the citation manager software, or some other information in parentheses in German. As an English reader, I cannot tell whether this should be informational text or not. Please revisit and clarify.

Methods

5) It would increase transparency to include additional details around recruitment such as: How many emails did you send out? What was the approx. response rate? How many participants were acquired through snowball sampling?

6) Considering that recruitment was within the researcher's network or snowballed, did you take any measures to protect against contamination between participants who may know each other and may have discussed the interview with someone who had not interviewed yet? This could've led future participants to reflect on or seek more information on smoking and MS and altered the knowledge outcome.

7) Similarly, how far in advance of the interview did participants do the online survey with questions asking about their knowledge? What protections/instructions were in place to help reduce the risk of bias introduced by participants looking up information between the two data collection points? The authors even noted a discrepancy between lower survey knowledge and higher interview knowledge. If the interview was not immediately after the survey, the risk of bias should be noted in the limitations.

8) It would be helpful (and likely bolster rigor) to know how the quotes and interview guide were translated and if they were audited by a second person for fidelity to the original meaning.

9) It is unclear why passive smoking (a risk factor) and NRTs (an intervention) are in the same sub-theme. Could you please elaborate so that readers can follow what the authors intended.

Results

10) It would be helpful if the number of subthemes was clearly listed similar to the number of main themes. This could also be an opportunity for a figure. Otherwise, readers have to count the paragraphs under each theme and cross reference the table, assuming all sub-themes are listed in the table but not having been explicitly informed of such.

11) It would greatly enhance transparency and the reader's ability to interpret or build upon this work if the actual results were specified instead of using four quartiles (i.e., <25%, 26% - 50%, 51% - 75%, >75%). For example, readers may be interested in using X method if 25% (n=3) of participants supported it, but less likely if only 8% (n=1). However, the results report this all under "a few".

12) Line 289 – Two participants noted having ample time. Can authors please elaborate on any similarities that could inform future work? For example, if they worked the same setting, like rehab, or private practice? Or was it two nurses who may have more time with the patients? These insights could make this work more impactful and help drive the delivery of future interventions. Any additional insights should likely have a corresponding discussion point in the discussion/future research section.

Additional points - throughout

1) This work would greatly benefit from a thorough editorial process as there are many areas which may be distracting for readers. For example, the results section has many sentences with awkward phrasing and inappropriate utilization of commas. Additionally, clarity and reader comprehension could be improved by specifying who/what is being discussed instead of relying so heavily on “they” “their” “them”. For example, line 261 – 263 (included below) puts a high cognitive burden on readers and could be misinterpreted (e.g., when who is interested? Patients or clinicians?).

“Only a few said, that they talk about NRTs with their patients when they are interested, while a few others don’t mention them because they are unsure about their effectiveness und usefulness.”

Could be clearer with something like: “A few clinicians reported talking about NRTs with patients who are interested. Conversely, clinicians who were unsure about NRT effectiveness (n= #) did not discuss this treatment option.”

2) Wherever possible, editing “smokers” to something like “people who smoke” “ PwMS who smoke” would be more aligned with the current shift towards using “people first” language.

VERSION 1 - AUTHOR RESPONSE

#1 Response to Reviewer 1

Thank you very much for your kind feedback and your comments. Please find our replies below.

Comment 1.1: Line 25: Define “pwMS”

Reply 1.1*: We added the definition und put the abbreviation in parentheses. It now reads:*

[Changes in the manuscript: page 2, line 25] *“[...] Smoking cessation promotion practices of MS clinicians are not meeting the needs of people with MS (pwMS). [...]”*

Comment 1.2: Line 163: Add “us” after “allow”

Reply 1.2*: We have done as you suggested. The new sentence now reads:*

[Changes in the manuscript: page 8, line 180] *“[...] but stays on the surface level, which allows us to describe the data of interest in detail [...]”.*

Comment 1.3: Lines 179, 253, 261, 265, 277, 278, 299: Remove the comma before the “that”

Reply 1.3*: We checked and improved the whole manuscript for English language and grammar and removed the commas that you found as well as some extra ones, which we found.*

Comment 1.4: Lines 301-303: The sentence has some grammatical errors and needs more clarification regarding "social aspects."

Reply 1.4: *We revised the whole sentence to correct errors and to improve clarity:*

[Changes in the manuscript: page 16, lines 332-338] *"~~Other mentioned aspects in this matter where the perceived patients' lack of acceptance and willingness to deal with this topic and the difficulty to change a behaviour that often has a lot social aspects attached to it.~~ Other aspects mentioned in this matter were the perceived lack of patients' acceptance and willingness to address the topic. Participants also said that patients often explain that smoking is closely linked to social settings, including daily routines or peer relationships, which makes quitting particularly difficult.*

#2 Response to Reviewer 2

Thank you very much for your valuable feedback and comments. Please find our replies below.

Comment 2.1: There is inconsistency while referencing interdisciplinary participants. The abstract clearly lists all participants as clinicians using parentheses, however, Table 1 then lists 8 clinicians vs 4 nurses. This introduces confusion in results when authors report things such as "only a few of the clinicians" because readers don't clearly know the denominator (e.g., 8 or 12?). I suggest being consistent throughout and when referencing only physicians, to use a more specific term like physician or neurologist to improve clarity.

Reply 2.1: *Good point. Generally, when we mention "clinicians" we mean both neurologists and nurses. We therefore changed the wording in Table 1, which now lists "Neurologist" and "Nurse" instead of "Clinician" and "Nurse". When analysing our interviews, we found that there were no considerable differences between answers from these two groups. Therefore, in the Results we mostly refer to "clinicians". With the changes in Table 1 it should now be clear that we mean both groups when referring to "clinicians". To further improve clarity in this matter, we also revised a respective sentence in Section 3.1:*

[Changes in the manuscript: page 10, line 216] *"All interviews lasted between 15 and 25 minutes, with a mean duration of 20 minutes. We interviewed 12 clinicians: eight neurologists and four MS nurses. Three out of all twelve… […]"*

Comment 2.2: Authors mention four themes and only report results on three. While the 4th can be somewhat inferred from the barriers, it would be more complete and balanced to clearly state the reported needs and wishes. Additionally, the last sentence of the results appears to report on theme 2 (current practices), but is listed under theme 3 (barriers). It would be clearer to list results together by theme, if this sentence is included after revisions, it should be moved up.

[Changes in the manuscript: page 2, line 32-47] ***Reply 2.2***: *We agree and revised the section in the abstract:*

"We identified 27 codes across four themes: 1) Knowledge: The 12 participants demonstrated a satisfactory general knowledge of the negative impacts of smoking on MS. 2) Current practice: Significant variability was reported in the current practices, with some clinicians providing detailed advice while others merely assessing smoking status without further advice or assistance. 3) Barriers: Key barriers identified included limited consultation time, perceived lack of patient motivation, and insufficient availability of resources, like information material, for effective smoking cessation support. 4) Needs and wishes: Participants wished for specific smoking cessation courses to which they could refer patients, as well as information material to use during patient counseling."

Comment 2.3: It appears that there is more than a slight risk of selection bias from using purposive/judgement sampling which relies on the judgement of researchers and is subject to additional (subconscious, conscious) bias. To improve transparency, this risk should just be openly acknowledged and "slight" should be removed. While this is also listed in the limitations, there is no mention of additional diversity of participants and particularly race or ethnicity. If future work could improve upon this work by broadening to a more diverse sample, it should be noted in the discussion or limitations.

Reply 2.3*: We agree and therefore removed the "slight" in the respective sentence. We also added a sentence in the Limitations to address the topic of a more diverse sample:*

[Changes in the manuscript: page 22, lines 452-454] *"[...] But we expect this effect to not be significant, as type of facility, geographical location and other participant characteristics are still well distributed. <u>Still, future studies might want to consider to include more sample characteristics (e.g. ethnicity) to further ensure high heterogeneity among participants or to use a different sample technique</u>."*

Comment 2.4: Introduction
4) Line 82 – This may have a citation that was not included in the citation manager software, or some other information in parentheses in German. As an English reader, I cannot tell whether this should be informational text or not. Please revisit and clarify.

Reply 2.4*: This is the translation of "the Association of the Scientific Medical Societies". But we understand that this might be confusing to non-German readers and reduced the information in the parenthesis to its abbreviation. We also added a source to the site from the Association. It now reads:*

[Changes in the manuscript: page 5, line 92] *"Additionally, the Association of the Scientific Medical Societies in Germany (~~Arbeitsgemeinschaft der Wissenschaftlichen Medizinischen Fachgesellschaften e. V.,~~ AWMF, (21)) recommends to all [...]"*

Comment 2.5: Methods
5) It would increase transparency to include additional details around recruitment such as: How many emails did you send out? What was the approx. response rate? How many participants were acquired through snowball sampling?

Reply 2.5*: To increase transparency, we included the information in Section 2.2:*

[Changes in the manuscript: page 6, lines 121-129] *"In order to recruit MS experts from different healthcare settings (hospitals, rehabilitation facilities and neurology practices) and occupations (neurologist, MS nurse), we purposefully chose and used connections from the broad German network of our working group, to get in first contact with some of our participants. ~~After this initial start, we could then use a snowball system, by asking each participant for contact information for further possible participants~~. In the period from April to July 2023, we initially approached 14 possible participants in total ~~everyone~~ via email and ~~initially~~ recruited 11 participants of them ~~in the period from April to July 2023~~. Two participants did not respond in the first place and one further participant stopped responding to our emails before an interview could be conducted. In addition to our initial start, we also used a snowball system, by asking each participant for contact information for further possible participants. Through this, we managed to include one more participant. Hence, we recorded and analysed a total of 12 interviews."*

Comment 2.6: Considering that recruitment was within the researcher's network or snowballed, did you take any measures to protect against contamination between participants who may know each other and may have discussed the interview with someone who had not interviewed yet? This could've led future participants to reflect on or seek more information on smoking and MS and altered the knowledge outcome.

Reply 2.6*: As described in Reply 2.5 we only recruited one participant via snowball sampling. Here, we cannot rule out the contamination that you have described. For all other participants, however, we judge this risk to be very low, as they are from different facilities in many different cities. In the added sentence from Reply 2.3 we now suggest that future studies might want to consider using a different sampling technique. We hope, that these adjustments suffice. Please let us know, if you think, that we should emphasize this aspect even more.*

Comment 2.7: Similarly, how far in advance of the interview did participants do the online survey with questions asking about their knowledge? What protections/instructions were in place to help reduce the risk of bias introduced by participants looking up information between the two data collection points? The authors even noted a discrepancy between lower survey knowledge and higher interview knowledge. If the interview was not immediately after the survey, the risk of bias should be noted in the limitations.

Reply 2.7*: This is a valid point. Unfortunately, we did not protect against this possible bias. We added a sentence in our Limitations, to acknowledge this:*

[Changes in the manuscript: page 23, lines 458-461] *"Finally, in some instances, a delay of one or two days occurred between the completion of the questionnaire and the interview. This could have introduced a potential bias, as we were unable to control whether participants informed themselves about the topic in the meantime."*

Comment 2.8: It would be helpful (and likely bolster rigor) to know how the quotes and interview guide were translated and if they were audited by a second person for fidelity to the original meaning.

Reply 2.8*: We added the respective information:*

[Changes in the manuscript: page 7, lines 137-140] *"The interviews were held in German and all quotes in this paper were translated into English in retrospect using the translation software DeepL Additionally, all quotes were double checked for fidelity by the main author and for comprehensiveness by all authors."*

Comment 2.9: It is unclear why passive smoking (a risk factor) and NRTs (an intervention) are in the same sub-theme. Could you please elaborate so that readers can follow what the authors intended.

Reply 2.9*: Because both topics were seldomly mentioned, they ended up in the same sub-theme in our original coding system. We agree, however, that it makes more sense to the reader if we divide them into separate sub-themes. We therefore gave each topic now its own heading. We also made sure to include exemplary quotes for both sub-themes in Table 3.*

Comment 2.10: It would be helpful if the number of subthemes was clearly listed similar to the number of main themes. This could also be an opportunity for a figure. Otherwise, readers have to count the paragraphs under each theme and cross reference the table, assuming all sub-themes are listed in the table but not having been explicitly informed of such.

Reply 2.10*: Good point. We included a new Figure 1 in the manuscript, which gives an overview of the themes and their respective sub-themes, as well as a sentence in section 3.2 that introduces the figure:*

[Changes in the manuscript: page 11, lines 227-229] *"Following, each theme and its sub-themes will be explored in detail and quotes from the interviews will be provided in separate tables. Figure 1 provides an overview of all four themes and their respective sub-themes."*

Comment 2.11: It would greatly enhance transparency and the reader's ability to interpret or build upon this work if the actual results were specified instead of using four quartiles (i.e., <25%, 26% - 50%, 51% - 75%, >75%). For example, readers may be interested in using X method if 25% (n=3) of participants supported it, but less likely if only 8% (n=1). However, the results report this all under "a few".

Reply 2.11*: That is a good aspects, which we did not consider. So, to increase transparency and the ability to interpret our results, we followed your suggestion and added the number of participants in parenthesis throughout the Results section. We also removed the following sentence, as it is no longer necessary:*

[Changes in the manuscript: page 9, lines 204-207] ~~*" Further, to simplify the terms used to describe the proportions of participants reporting on certain topics, we defined the following*~~

~~categories: A few = less than 25 % of participants, some = 26-50 %, many = 51-75%, most > 75%, as has been applied before similarly (30).”~~

Comment 2.12: Line 289 – Two participants noted having ample time. Can authors please elaborate on any similarities that could inform future work? For example, if they worked the same setting, like rehab, or private practice? Or was it two nurses who may have more time with the patients? These insights could make this work more impactful and help drive the delivery of future interventions. Any additional insights should likely have a corresponding discussion point in the discussion/future research section.

Reply 2.12*: When writing the results, we checked if specific answers were given only by certain groups of participants (e.g. only by nurses, or only by those working in a rehab facility). However, we only found one aspect, where this was the case. We mention this in Section 3.2.2 “Referrals”. Here, all clinicians working in a rehab facility would refer their patients to further cessation services, while the same was true for only one clinician working in a different type of facility. Our ability to cluster our findings like this was, of course, limited by the small sample size. But to explain this aspect of our findings, we added a sentence in our Discussion:*

[Changes in the manuscript: page 22, lines 437-438] *“While all our participants working in a rehabilitation facility offered referrals to smoking cessation services to the patients~~, the opposite was the case~~ it looked quite different for the participants working in a hospital setting or a neurology practice. […] But apart from that, we did not find any noteworthy differences based on profession or type of facility.”*

Comment 2.13: This work would greatly benefit from a thorough editorial process as there are many areas which may be distracting for readers. For example, the results section has many sentences with awkward phrasing and inappropriate utilization of commas. Additionally, clarity and reader comprehension could be improved by specifying who/what is being discussed instead of relying so heavily on “they” “their” “them”. For example, line 261 – 263 (included below) puts a high cognitive burden on readers and could be misinterpreted (e.g., when who is interested? Patients or clinicians?).

“Only a few said, that they talk about NRTs with their patients when they are interested, while a few others don’t mention them because they are unsure about their effectiveness und usefulness.”

Could be clearer with something like: “A few clinicians reported talking about NRTs with patients who are interested. Conversely, clinicians who were unsure about NRT effectiveness (n= #) did not discuss this treatment option.”

Reply 2.13*: Thank you for this comment and your suggestion. It is, of course, in our own interest to increase readability. We therefore revised several sentences throughout the whole document. Additionally, please see also comment and reply 1.3 and 1.4.*

[Changes in the manuscript: page 14, lines 286-290] *~~"Only a few said, that they talk about NRTs with their patients when they are interested, while a few others don't mention them because they are unsure about their effectiveness und usefulness."~~* → *"A few clinicians (n=3) reported discussing NRTs with patients who expressed interest. In contrast, clinicians who were uncertain about the effectiveness of NRTs (n = 2) did not bring up this treatment option."*

[Changes in the manuscript: page 12, line 242] *"They ~~could mention and~~ were able to describe, in varying levels of detail, the increased risk for MS onset and progression".*

[Changes in the manuscript: page 12, lines 249-252] *"Here~~, none of the participants~~ no clinician knew or used specific methods for smoking cessation promotion. ~~and~~ Only one participant explained that he would ~~use~~ follow smoking cessation treatment guidelines ~~and~~ by asking patients ~~for~~ about their smoking behaviour ~~as a part of MS specific treatment guidelines.~~*

[Changes in the manuscript: page 13, lines 263-265] *"One participant even ~~mentioned that they also~~ reported to encourage people who do not smoke ~~non-smokers~~ to not pick up smoking."*

[Changes in the manuscript: page 13, lines 267-268] *"Only ~~a few of the MS~~ two clinicians stated that they either don't talk about the topic with the patients at all or ~~only check for~~ simply assess the smoking status without providing more information about the negative consequences of smoking on MS."*

[Changes in the manuscript: pages 13-14, lines 271-278] *"Some (n=5) of the participants refer the patients to further smoking cessation assistance services like courses or seminars, or ~~give them a~~ provide flyers with ~~more information~~ details ~~about~~ on where ~~they can get more~~ to seek further help. This was ~~the case for~~ reported by all participants working in a rehabilitation facility, but only ~~the case for one participant~~ by one clinician working in a clinic. ~~All the other~~ The remaining participants who work in either a clinic, hospital or neurology practice stated, that they don't refer ~~their~~ patients to ~~smoking~~ cessation services or ~~give them more information on where they could go~~ provide additional resources."*

[Changes in the manuscript: page 14, lines 280-282] *"Only three participants ~~explained that they talk about~~ reported discussing the topic of passive smoking and MS with their patients. However, they mentioned that this wouldn't be part of a routine and therefore ~~wouldn't happen in all~~ ~~cases~~ occurred only occasionally.*

[Changes in the manuscript: page 14, line 292-299] *"Many (n=8) ~~of our~~ participants ~~stated, that smoking has a~~ acknowledged a high relevance of smoking in the field of MS and stated that it the topic of smoking and MS should be ~~talked about, especially with currently smoking patients~~ actively addressed. ~~According to them~~, They felt that ~~this topic~~ it should be an integral part, ~~when talking~~ of conversations with pwMS about ~~what they can do~~ strategies to slow down ~~the~~ progression and improve prognosis ~~of their diseases~~ beside medical treatments. Only a few clinicians (n=3) ~~said~~ considered ~~that~~ smoking ~~is not so important~~ as less significant compared to ~~at least when comparing it with~~ other topics ~~that come up when people have MS, leaving~~ placing it ~~therefore~~ in the "second row" of priority."*

[Changes in the manuscript: page 15, lines 302-309] *"Only one ~~of the~~ MS nurse~~s~~ said that she doesn't feel responsible to talk about smoking, as this would be the responsibility of the ~~clinicians~~ neurologists or physicians. A few (n=3) other clinicians additionally stated that, while they feel responsible to raise the topic of smoking and MS ~~when talking with patients~~ during consultations, ~~that~~ they don't see themselves as smoking cessation agents~~. This would be, according to them, a more fitting~~ In their view this responsibility ~~for the~~ is more appropriately placed with general practitioners."*

[Changes in the manuscript: page 16, lines 321-326] *"~~They~~ Participants highlighted ~~the number of different~~ the wide range of topics they need to ~~talk~~ address during consultations ~~about with patients, which compete in~~ many of which compete for priority. Consequently, our participants often find themselves unable to ~~go more deeply into~~ talk more about smoking-related matters due to the pressing need to address medication and therapy concerns foremost. Moreover, the substantial time demands of administrative and organizational duties ~~would~~ further ~~reduce~~ limited the time allocated for counseling activities."*

[Changes in the manuscript: page 17, lines 341-344] *"~~This is, f~~For example, ~~due to~~ clinicians expressed reluctance to raise the topic of smoking because they did not ~~the fact that they don't~~ want to be perceived as moralizing or acting as a ~~the~~ scapegoat ~~or upholder of morals~~."*

[Changes in the manuscript: page 18, lines 368-370] *"They ~~could show them, for instance, some~~ perceived that using concrete ~~numbers~~ figures or graphs ~~to better~~ for visualisation ~~and~~ could facilitate their talking points during counseling. "*

Comment 2.14: Wherever possible, editing "smokers" to something like "people who smoke" "PwMS who smoke" would be more aligned with the current shift towards using "people first" language.

***Reply 2.14**: Good point. We went through the whole manuscript and changed the wording accordingly.*

VERSION 2 - REVIEW

**Reviewer**	**2**
**Name**	**Polick, Carri S**
**Affiliation**	**Duke University**
**Date**	**20-Jun-2025**
**COI**	

Thank you for your detailed clarifying edits. I have no further suggestions and recommend this paper for publication.
